# Neoadjuvant Imatinib in Gastrointestinal Stromal Tumors (GIST): The First Analysis of a Mexican Population

**DOI:** 10.7759/cureus.65001

**Published:** 2024-07-20

**Authors:** Rafael Medrano Guzman, Xavier Lopez Lara, Atl Simon Arias Rivera, Luis E Garcia Rios, Moises Brener Chaoul

**Affiliations:** 1 Surgical Oncology, Centro Médico Nacional Siglo XXI, Mexico City, MEX; 2 General Surgery, Hospital Angeles Lomas, Huixquilucan, MEX; 3 Surgical Oncology, Hospital Angeles Lomas, Huixquilucan, MEX

**Keywords:** c-kit, pdgfra, surgical resection, imatinib, gist

## Abstract

Introduction

Gastrointestinal stromal tumors (GISTs) are neoplasms originating from the interstitial cells of Cajal, pacemaker cells responsible for intestinal motility. Patients with locally advanced GISTs and those with borderline resections due to the proximity of vital anatomical structures, which could result in unacceptable post-surgical morbidity, require special therapeutic consideration.

Imatinib, a tyrosine kinase inhibitor, has demonstrated significant success in the non-surgical management of metastatic GIST, and its favorable impact on overall survival in the adjuvant setting makes it logical to speculate on the benefit it could provide as a neoadjuvant medication in patients with locally advanced disease.

Methods

Patients aged 18-90 years with a diagnosis of GIST confirmed by immunohistochemistry (CD117 positivity) who were treated at the Oncology Hospital of Centro Médico Nacional Siglo XXI in Mexico City from January 2012 to December 2016 were included in the study. It is a retrospective study with a duration of four years. Clinical data were collected from the medical records, which included sex, age, tumor location, initial resectability, reason for unresectability, initial tumor size, and mitotic rate. In the case of unresectable disease, patients who were evaluated by medical oncology and who had received treatment with 400 mg of imatinib daily were evaluated.

Results

A total of 312 patients diagnosed with GIST were analyzed. One hundred thirty-one were men (42%) with a mean age of 57 years, and 181 were women (58%) with a mean age of 59 years. The most frequent anatomical location was the stomach (n=185, 59.2%). At the time of diagnosis, 210 patients (67.3%) presented with resectable disease, while n=102 patients (32.7%) had unresectable disease. A total of 102 patients with unresectable disease received therapy with 400 mg of imatinib per day. Sixteen patients (15.7%) presented a reduction in tumor dimensions and underwent surgery.

Conclusion

The study highlights the importance of complete surgical resection and the potential benefit of neoadjuvant imatinib therapy in converting unresectable to resectable disease. The results suggest that imatinib can be effective in converting unresectable GISTs to resectable ones, allowing for a complete resection to be performed and obtaining an R0 resection in 93.7% of these cases.

## Introduction

Gastrointestinal stromal tumors (GIST) are neoplasms that derive mainly from precursors of the interstitial cells of Cajal, pacemaker cells responsible for intestinal motility. However, GISTs with PDGFRA mutations can arise from telocytes, and BRAF-mutated GISTs can be derived from smooth muscle cells [1,2].

GISTs are the most common sarcomas, comprising a heterogeneous group of tumors with a variety of predominantly mutually exclusive oncogenic mutations, mainly in KIT and PDGFRA. Most GISTs present with gastrointestinal symptoms, which include bleeding (>50%), obstruction (35%), and pain (20%). Regarding their location, 60-65% of GISTs are gastric, and 20-25% are located in the small intestine; less frequently involved sites include the rectum (3-5%), colon (1-2%), and esophagus (1%) [3-13].

The incidence of these tumors varies from 6 to 22 cases per 1,000,000 individuals per year, varying by geographical region. GISTs are diagnosed at an average age of 65 years, although they can be diagnosed in any age group [14-17].

GISTs without KIT or PDGFRA mutations (wild-type GISTs) are more frequent in the pediatric population, while KIT-mutated GISTs are more commonly diagnosed in patients over 18 years of age. Although few studies have reported on the incidence of molecular alterations in GISTs, it is estimated that KIT mutations occur in eight cases per 1,000,000 individuals per year, while the incidence of PDGFRA mutations is <3 cases per 1,000,000 individuals per year [7-15,18].

Moreover, correlations between the anatomical site and specific mutations have been established: tumors with KIT exon 9 mutations predominantly occur in the small intestine, colon, or rectum. On the other hand, tumors with PDGFRA mutations, most commonly D842V, arise mainly in the stomach. Furthermore, GISTs with mutations in the succinate dehydrogenase complex (SDH) tend to be multifocal, although they commonly present as gastric tumors in young patients and display a predominance in women [16-18].

Although the majority of GISTs are sporadic, a high frequency of hereditary germline mutation-associated tumors has been described in certain families, a condition known as familial GIST syndrome [19,20].

Additionally, an association between GISTs and other pathological entities such as type 1 neurofibromatosis and other cancers has been established; the most common association is with genitourinary tumors. Clinical syndromes involving GISTs include Carney's triad, which is the association of a GIST with paraganglioma and a pulmonary chondroma, and Carney-Stratakis syndrome, which is defined as the coexistence of a paraganglioma and a gastric GIST [21].

Regarding prognosis, approximately 50% of patients present with metastatic disease upon diagnosis, the most commonly involved sites being the liver and peritoneum; bone and brain metastases are rare and occur very late in the course of the disease. Virtually all GISTs have the potential to recur following a complete surgical resection of the primary tumor. The five-year recurrence rate is greater than 50%, with a mean recurrence-free survival of 18 to 24 months [22-26].

The most frequent site of recurrence is the liver (67%), followed by the peritoneum. Patients who develop recurrent disease after initial surgical treatment present resectability in 26-30% of cases. The five-year survival rate is 35-65%. In patients with unresectable disease, median survival is 10-20 months [27-28].

Patients with locally advanced GIST and those with borderline resections due to the proximity of vital anatomical structures, which could result in unacceptable post-surgical morbidity, require special therapeutic consideration. The marked success that the tyrosine kinase inhibitor imatinib has demonstrated in the non-surgical management of metastatic GIST, as well as its favorable impact on overall survival in the adjuvant setting, makes it logical to speculate on the benefit it could provide as a neoadjuvant medication in patients with locally advanced disease [29-32].

Fiore et al. published one of the largest single-institutional case series of locally advanced GIST, which included a total of 15 patients who received neoadjuvant therapy and had a surgical resection subsequently. In this study, 14 of the 15 cases obtained at least some evidence of a radiological response with the use of neoadjuvant imatinib and achieved a three-year disease-free survival rate of 77% in high-risk groups. Furthermore, Blesus et al. analyzed a subgroup of patients with locally advanced and metastatic GIST from BFR14, a phase III study, who were randomized to interruption vs. continuation of imatinib. Twenty-five patients in this study had locally advanced GIST; 60% responded to treatment with imatinib, and nine underwent surgical resection, achieving a three-year progression-free survival and overall survival of 67% and 89%, respectively [33-35].

On the other hand, patients who did not undergo surgical resection had similar outcomes compared to those of patients with metastatic disease. In summary, the usefulness and benefit of performing a complete resection of GIST have been demonstrated, although studies concerning unresectable GISTs are few and limited to small series. A potential benefit has been observed in the use of imatinib for the conversion of unresectable to resectable GIST, potentially achieving similar survival when complete resection is achieved. Here, we describe the rate of conversion of unresectable to resectable disease following the administration of imatinib in patients with GIST who were treated in the Oncology Hospital of Centro Médico Nacional Siglo XXI in Mexico City [34,35].

## Materials and methods

Inclusion criteria: Patients between 18 and 90 years old with a diagnosis of GIST confirmed by immunohistochemistry (established as CD117 positivity) who were treated at the Oncology Hospital of Centro Médico Nacional Siglo XXI in Mexico City from January 2012 to December 2016. 

It is a retrospective study with a duration of four years. Clinical data were collected from the medical records, which included sex, age, tumor location, initial resectability, reason for unresectability, initial tumor size, and mitotic rate.

In the case of unresectable disease, patients who were evaluated by medical oncology and who had received treatment with 400 mg of imatinib daily were evaluated.

Exclusion criteria: Patients who were deemed non-compliant with the treatment scheme were excluded from the study.

Quantitative variables were evaluated, variables with a normal distribution were described as means, and variables with a free distribution were described as medians and interquartile ranges. Statistical analysis was performed using IBM Corp. Released 2016. IBM SPSS Statistics for Windows, Version 24.0. Armonk, NY: IBM Corp. and Microsoft Excel 2010.

## Results

A total of 312 patients diagnosed with GIST were analyzed. One hundred thirty-one were men, n=131 (42%) with a mean age of 57 years, and n=181 were women (58%) with a mean age of 59 years. The mean tumor size was 9.5 cm. In patients with initially resectable disease, the mean tumor size was 7.8 cm; the mean tumor size of initially unresectable tumors was 13.2 cm. Tumors with a low mitotic index (less than 5 mitoses per 50 HPF) accounted for n=163 (52.2%) of all cases. The mitotic index could not be determined in 26.6% of the cases due to an omission in the histopathological report. In patients with initially resectable disease, most tumors displayed a low mitotic index, representing n=60 (19.52%) of cases, followed by tumors with a medium mitotic index (6 to 10 mitosis/50 HPF), which represented n=60 (19.52%). Tumors with a high mitotic index (over 10 mitoses/50 HPF) represented n=10 (3.33%), while those not specified in the histopathological report were n=7 (2.38%). In patients with initially unresectable disease, tumors with a medium mitotic index predominated, accounting for n=42 (13.72%) of cases, followed by tumors with a low mitotic index, which represented n=18 (5.88%) of cases, while patients with a high mitotic index represented n=12 (3.92%). No mitotic index in the histopathological report was found in n=238 (76.47%) of patients. Patient and tumor characteristics are further detailed in Table 1.

**Table 1:** Patient and tumor characteristics The number of cases is presented as N, the percentages are expressed as %, the age is expressed as years, and the tumor size is expressed as cm. p-value is considered significant (p<0.05).

Category	Number of Cases	Percentage (%)	Average Age (Years)	Mean Tumor Size (cm)
Total Population	312	100%	58	NA
Men	131	42%	57	NA
Women	181	58%	59	NA
Tumor Location				
Gastric	185	59.29%	NA	NA
Small Bowel	98	31.41%	NA	NA
Colon	6	1.92%	NA	NA
Rectum	2	0.64%	NA	NA
Duodenum	11	3.52%	NA	NA
Intra-Abdominal (Unspecified Site)	10	3.20%	NA	NA
Resectability				
Initially Resectable	210	67.30%	NA	NA
Initially Unresectable	102	32.69%	NA	NA
Mitotic Rate (Mitosis/50 CGA)				
0 - 5	163	52.20%	NA	NA
6 - 10	55	17.62%	NA	NA
> 10	11	3.52%	NA	NA
Unspecified	83	26.60%	NA	NA
Tumor Size (Mean in Centimeters)				
Total	NA	NA	NA	9.5
Resectable	NA	NA	NA	7.78
Unresectable	NA	NA	NA	13.2

**Figure 1:**
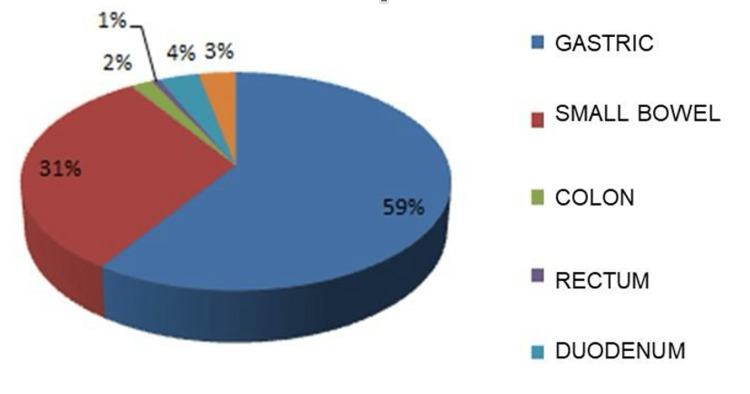
Tumor location The different types of tumor location are expressed in %. p-value is considered significant (p<0.05).

**Figure 2:**
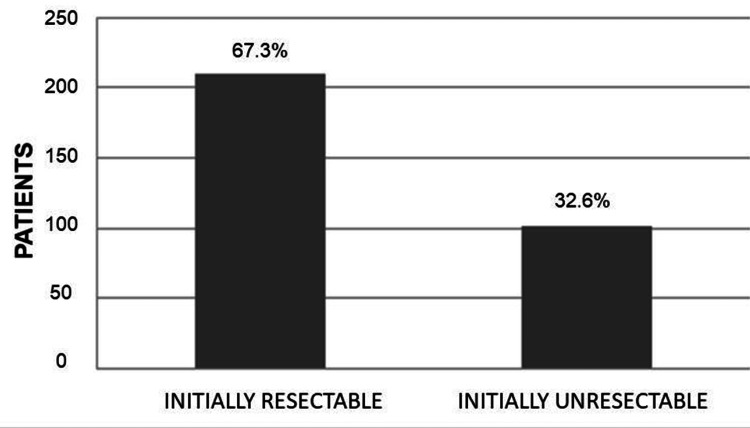
Initial resectability The number of cases is presented as N, and the percentages are expressed as %. p-value is considered significant (p<0.05).

**Figure 3:**
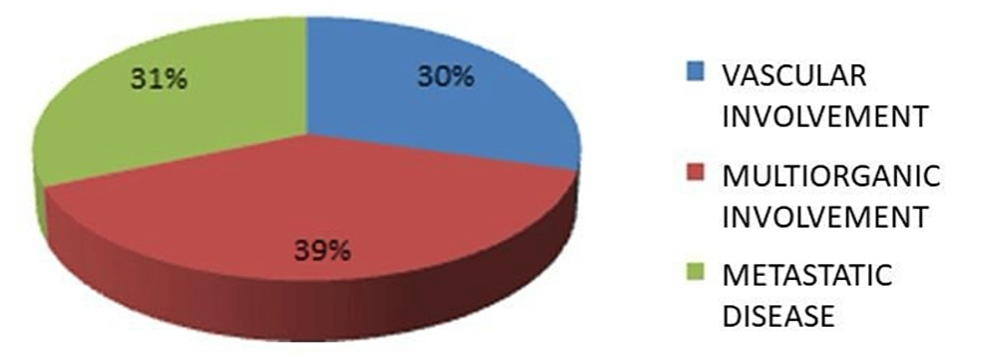
Reason for unresectability The data is expressed as %. p-value is considered significant (p<0.05).

**Figure 4:**
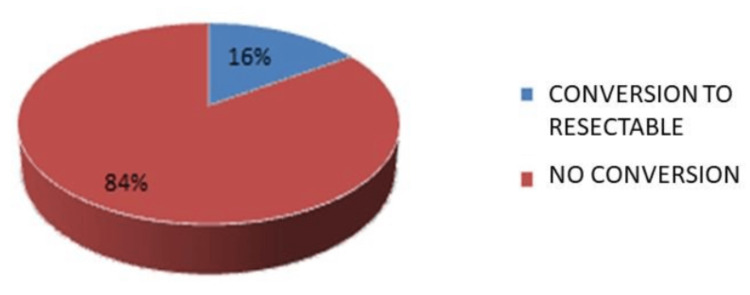
Conversion rate The data is expressed as %. p-value is considered significant (p<0.05).

The most frequent anatomical location was the stomach (n=185, 59.2%), followed by the small intestine (n=98, 31.4%), duodenum (n=11, 3.5%), colon (n=6, 1.9%), and rectum (n=2, 0.6%). N=10 cases (3.2%) with an intra-abdominal location without a specific site of origin were reported (Figure 1). At the time of diagnosis, 210 patients (67.3%) presented with resectable disease, while n=102 patients (32.7%) had unresectable disease (Figure 2). Reasons for unresectability included multi-organ involvement n=122 (39.2%), metastatic disease n=97 (31.3%), and vascular involvement n=91 (29.4%) (Figure 3). A total of 102 patients with unresectable disease received therapy with 400 mg of imatinib per day. Sixteen patients (15.7%) presented a reduction in tumor dimensions within six months and underwent surgery after the confirmation of tumor reduction by computed tomography with intravenous contrast. The mean time to conversion was six months from the start of neoadjuvant treatment (Figure 4). In the group of patients who did not achieve conversion, three patients were switched from their treatment regimen due to disease progression, six patients had their imatinib dose increased to 800 mg/day, and six patients presented severe treatment-related toxicity (three patients developed gastrointestinal bleeding, one patient developed thromboembolic disease, and two patients presented hepatotoxicity), of which four had their treatment suspended. Four patients died during drug administration. Of the remaining 64 patients (20.51%), six (1.92%) presented disease progression, and 58 (18.58%) had stable disease. In patients with initially resectable tumors who underwent surgical intervention, an R0 resection was achieved in n=304 (97.6%), while n=7 (2.38%) presented microscopic evidence of neoplastic cells in their margins (R1). No cases were reported as R2. In patients who presented conversion from unresectable to resectable disease, an R0 resection was achieved in 15 patients (4.8%), and an R1 resection was obtained in one patient (0.32%) (Table 2). The large tumor size being above 10 cm, the tumor invading surrounding organs or critical blood vessels, and the presence of metastases were considered unresectable factors. 

**Table 2:** Patients with a conversion from unresectable to resectable disease The number of patients is presented as N, and the percentages are expressed as %. p-value is considered significant (p<0.05).

Margins	Number of Patients	
Initially Resectable		
R0	205	97.61%
R1	5	2.38%
Initially unresectable with conversion		
R0	15	93.75%
R1	1	6.25%

## Discussion

The most common mutations in GISTs occur in KIT (60-70%) or PDGFRA (10-15%), although approximately 15% of these tumors are not KIT- or PDGFRA-mutated but are rather associated with other genetic alterations such as mutations in SDH, RAS, BRAF, the NF1 gene family, or even rarer ones such as gene fusions involving NTRK3 or FGFR1 [36-41].

It is estimated that 95% of GISTs are CD117 positive (CD117 is a product of the KIT 4q12 gene, type III receptor tyrosine kinase), generally due to the presence of activating mutations. These mutations affect regions that encode functional domains of the receptor, the most frequent mutations being located in exon 11, which encodes for the juxtamembrane domain (66-71%), as well as in exon 9 (10-20%) and exons 13, 14, and 17 (1% each). Of the mutations found in exon 11, they are most frequently located between codons 550 and 579, particularly in codons 557-559. Mutations in exon 9 (extracellular domain) have been identified at residues 502-503 and are associated with a high malignant potential [42].

Mutations in KIT are predictive of response to therapy, specifically imatinib. Patients with mutations in exon 11 display a better response, measured as overall survival, compared to those patients with mutations in exon 9 or the wild-type phenotype. To date, the most effective treatment for localized primary GISTs remains a complete surgical resection. Following primary resection, the five-year disease-free survival is 96% in low-risk GIST patients, 54% in intermediate-risk patients, and 20% in high-risk patients. The mean time for recurrence is 19-25 months [43,44].

It is estimated that approximately 5% of GISTs are CD117-negative, although 30-50% of these tumors harbor mutations in KIT, which may have therapeutic implications. The notion that CD117-negative tumors are mutation-negative is not entirely clear.

In the population included in this study, we describe that the most frequent anatomical site in GISTs was the gastric (59%), followed by the small intestine (31%). An undetermined intraabdominal location was reported in 3.2% of cases, which corresponds to mesenteric, epiploic, or retroperitoneal locations. In essence, our results were similar to those reported in the literature. At presentation and initial diagnosis, 67.3% of patients were determined to have resectable disease and were thus candidates for initial surgical management, while 32.6% of patients were classified as having unresectable disease. This finding is discordant with the results of a study published by Schwameis et al., where 90% of patients were found to have initially resectable disease at the time of diagnosis. We theorize that this discrepancy could be attributed to earlier diagnoses in developed countries. The most frequent cause for which patients were initially deemed unsuitable for resection was multiple organ involvement (39.2%), followed by metastatic disease (31%). In patients who had initially unresectable disease treated with imatinib, 15.7% presented conversion, allowing a complete resection to be performed and obtaining tumor-free resection margins in 93.7% of these cases. This is similar to the findings of a study published by Rutkowski et al., where 24 patients with unresectable GIST were treated with imatinib and a conversion rate of 17% was achieved, obtaining tumor-free resection margins in 83% of cases [45].

Furthermore, Rutkowski et al. conducted a trial in 10 EORT-STBSG centers, where 161 patients with non-metastatic, locally advanced GIST were treated with imatinib (400 mg/day). In this study, 8% of patients demonstrated a response to neoadjuvant imatinib, while 18.6% presented stable disease. An R0 resection was achieved in 83% of cases, and 37 cases presented disease recurrence in an average of 46 months. An overall survival of 87% and a disease-specific survival of 95% at five years were reported [45].

In summary, the benefit of imatinib administration in patients with initially unresectable GIST has been demonstrated and is well established, as has the impact on survival when tumor-free resection margins are achieved following neoadjuvant therapy.

In this study, which evaluated patients with a diagnosis of GIST treated at the Oncology Hospital of Centro Médico Nacional Siglo XXI in Mexico City, we demonstrate that the conversion rate of initially unresectable to resectable disease following neoadjuvant therapy with imatinib is 16%, which is consistent with the response and resectability rates for initially unresectable GIST reported in the literature. To the best of our knowledge, this is the first study in Mexico that reports the conversion rates of patients who are given neoadjuvant therapy with imatinib. Importantly, we report that an R0 resection was obtained in most cases, therefore improving the prognosis of these patients substantially.

The limitation of this study is the number of people in our article; a larger population needs to be recruited.

## Conclusions

In conclusion, Imatinib is an adequate treatment for the conversion of unresectable to resectable tumors trying to achieve posteriorly an R0 resection with surgical intervention. This study serves as a basis for prospective studies that will expand our knowledge about this therapeutic modality and thus represent a step closer to achieving the standardization of guidelines regarding the use of imatinib as a neoadjuvant therapy in patients with GIST. 
